# Supplementary material for: Evolutionary Genomics of Transposable Elements in Saccharomyces cerevisiae
Source: PLoS One. 2012 Nov 30;7(11):e50978. doi: 10.1371/journal.pone.0050978 (PMC3511429; doi:10.1371/journal.pone.0050978)
Supplement: File S8 — Visualization of recombination breakpoints in Ty1, Ty1/2 and Ty2 complete LTR sequences. The two approximate recombination breakpoints are highlighted with black lines. Asterisks highlight columns in the alignment where the base is conserved across all sequences. (PDF) [file pone.0050978.s008.pdf]

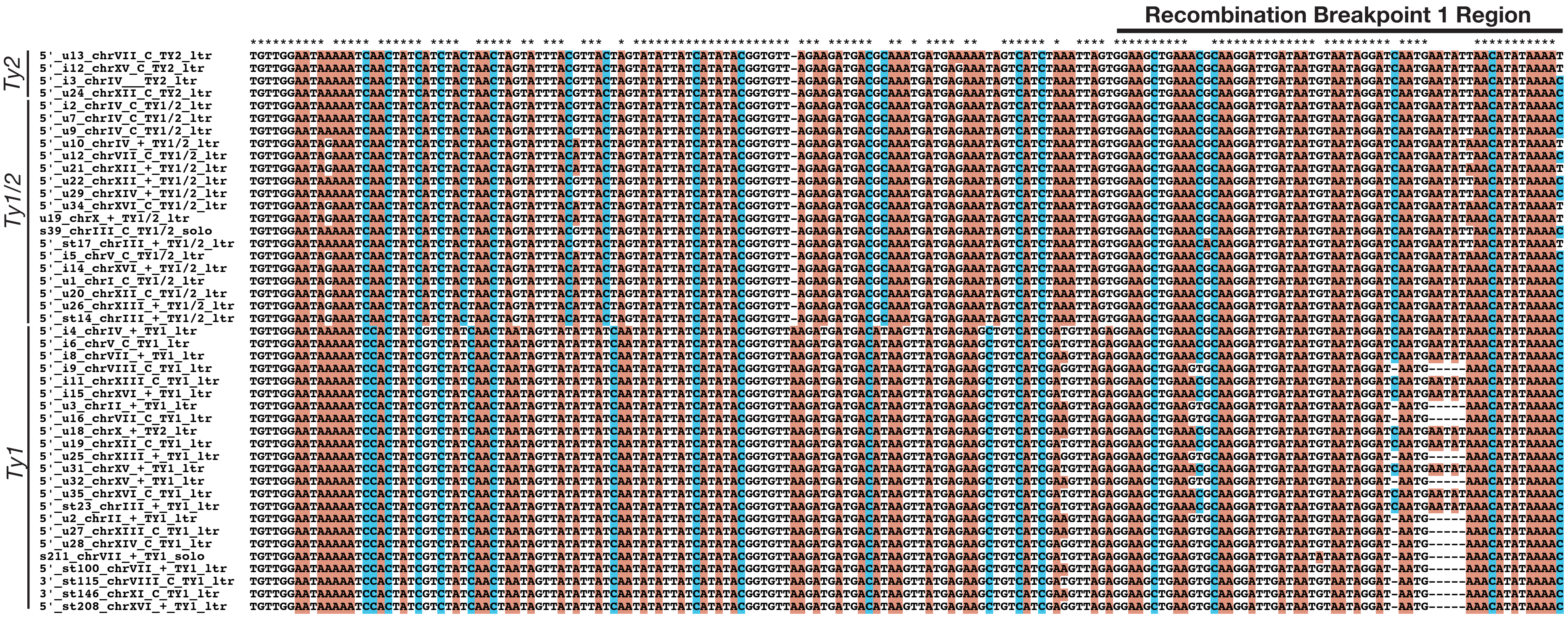
Recombination Breakpoint 1 Region
Ty2
Ty1/2
Ty1
5'_u13_chrVII_C_TY2_ltr
5'_i12_chrXV_C_TY2_ltr
5'_i3_chrIV__TY2_ltr
5'_u24_chrXII_C_TY2_ltr
5'_i2_chrIV_C_TY1/2_ltr
5'_u7_chrIV_C_TY1/2_ltr
5'_u9_chrIV_C_TY1/2_ltr
5'_u10_chrIV_+_TY1/2_ltr
5'_u12_chrVII_C_TY1/2_ltr
5'_u21_chrXII_+_TY1/2_ltr
5'_u22_chrXII_+_TY1/2_ltr
5'_u29_chrXIV_+_TY1/2_ltr
5'_u34_chrXVI_C_TY1/2_ltr
u19_chrX_+_TY1/2_ltr
s39_chrIII_C_TY1/2_solo
5'_st17_chrIII_+_TY1/2_ltr
5'_i5_chrV_C_TY1/2_ltr
5'_i14_chrXVI_+_TY1/2_ltr
5'_u1_chrI_C_TY1/2_ltr
5'_u20_chrXII_C_TY1/2_ltr
5'_u26_chrXIII_+_TY1/2_ltr
5'_st14_chrIII_+_TY1/2_ltr
5'_i4_chrIV_+_TY1_ltr
5'_i6_chrV_C_TY1_ltr
5'_i8_chrVII_+_TY1_ltr
5'_i9_chrVIII_C_TY1_ltr
5'_i11_chrXIII_C_TY1_ltr
5'_i15_chrXVI_+_TY1_ltr
5'_u3_chrII_+_TY1_ltr
5'_u16_chrVII_C_TY1_ltr
5'_u18_chrX_+_TY2_ltr
5'_u19_chrXII_C_TY1_ltr
5'_u25_chrXIII_+_TY1_ltr
5'_u31_chrXV_+_TY1_ltr
5'_u32_chrXV_+_TY1_ltr
5'_u35_chrXVI_C_TY1_ltr
5'_st23_chrIII_+_TY1_ltr
5'_u2_chrII_+_TY1_ltr
5'_u27_chrXIII_C_TY1_ltr
5'_u28_chrXIV_C_TY1_ltr
s211_chrVII_+_TY1_solo
5'_st100_chrVII_+_TY1_ltr
3'_st115_chrVIII_C_TY1_ltr
3'_st146_chrXI_C_TY1_ltr
5'_st208_chrXVI_+_TY1_ltr

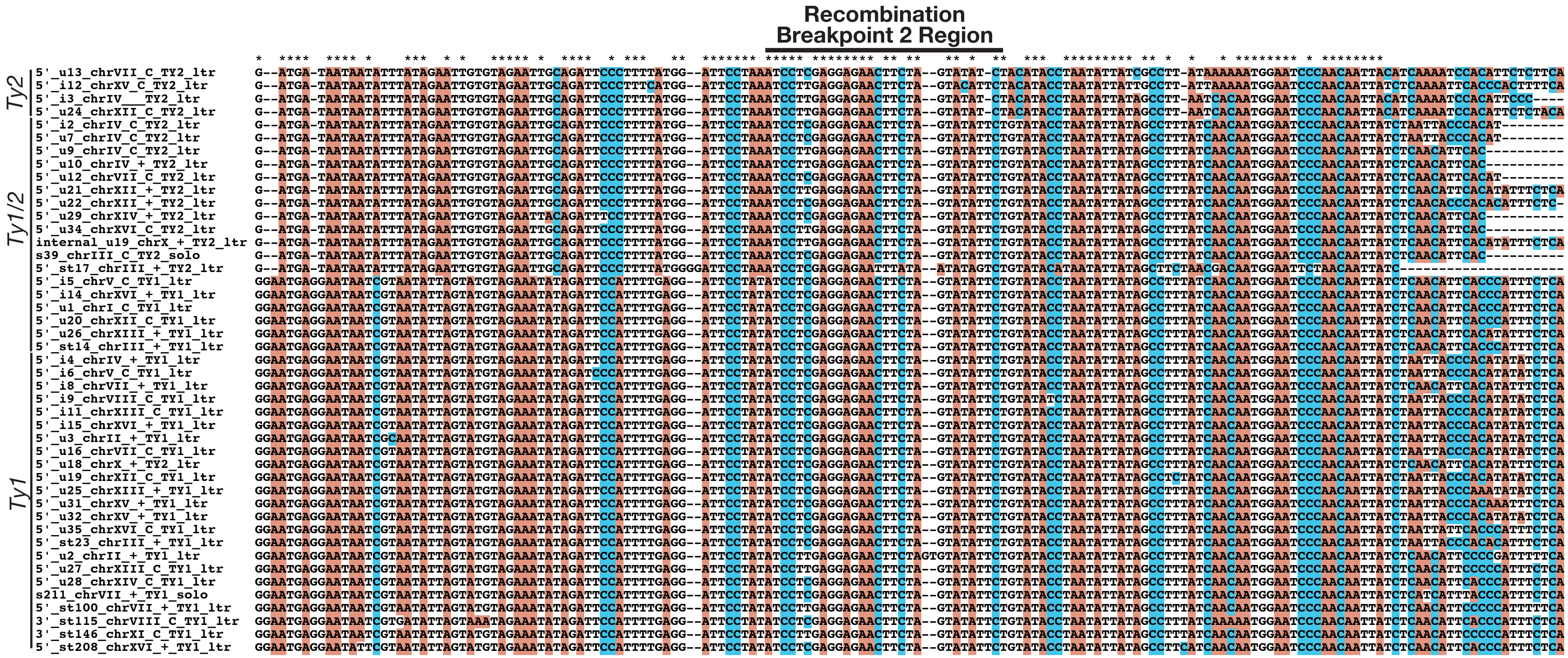

Recombination
Breakpoint 2 Region
Ty2
Ty1/2
Ty1
5'_u13_chrVII_C_TY2_ltr
5'_i12_chrXV_C_TY2_ltr
5'_i3_chrIV___TY2_ltr
5'_u24_chrXII_C_TY2_ltr
5'_i2_chrIV_C_TY2_ltr
5'_u7_chrIV_C_TY2_ltr
5'_u9_chrIV_C_TY2_ltr
5'_u10_chrIV_+_TY2_ltr
5'_u12_chrVII_C_TY2_ltr
5'_u21_chrXII_+_TY2_ltr
5'_u22_chrXII_+_TY2_ltr
5'_u29_chrXIV_+_TY2_ltr
5'_u34_chrXVI_C_TY2_ltr
internal_u19_chrX_+_TY2_ltr
s39_chrIII_C_TY2_solo
5'_st17_chrIII_+_TY2_ltr
5'_i5_chrV_C_TY1_ltr
5'_i14_chrXVI_+_TY1_ltr
5'_u1_chrI_C_TY1_ltr
5'_u20_chrXII_C_TY1_ltr
5'_u26_chrXIII_+_TY1_ltr
5'_st14_chrIII_+_TY1_ltr
5'_i4_chrIV_+_TY1_ltr
5'_i6_chrV_C_TY1_ltr
5'_i8_chrVII_+_TY1_ltr
5'_i9_chrVIII_C_TY1_ltr
5'_i11_chrXIII_C_TY1_ltr
5'_i15_chrXVI_+_TY1_ltr
5'_u3_chrII_+_TY1_ltr
5'_u16_chrVII_C_TY1_ltr
5'_u18_chrX_+_TY2_ltr
5'_u19_chrXII_C_TY1_ltr
5'_u25_chrXIII_+_TY1_ltr
5'_u31_chrXV_+_TY1_ltr
5'_u32_chrXV_+_TY1_ltr
5'_u35_chrXVI_C_TY1_ltr
5'_st23_chrIII_+_TY1_ltr
5'_u2_chrII_+_TY1_ltr
5'_u27_chrXIII_C_TY1_ltr
5'_u28_chrXIV_C_TY1_ltr
s211_chrVII_+_TY1_solo
5'_st100_chrVII_+_TY1_ltr
3'_st115_chrVIII_C_TY1_ltr
3'_st146_chrXI_C_TY1_ltr
5'_st208_chrXVI_+_TY1_ltr
